# Cryptocurrency volatility markets

**DOI:** 10.1007/s42521-021-00037-3

**Published:** 2021-08-02

**Authors:** Fabian Woebbeking

**Affiliations:** Goethe University Frankfurt, Frankfurt, Germany

**Keywords:** Cryptocurrency, Blockchain, Bitcoin, Volatility, Derivatives, Options, Liquidity, C5, F31, G1, G2

## Abstract

By computing a volatility index (CVX) from cryptocurrency option prices, we analyze this market’s expectation of future volatility. Our method addresses the challenging liquidity environment of this young asset class and allows us to extract stable market implied volatilities. Two alternative methods are considered to compute volatilities from granular intra-day cryptocurrency options data, which spans over the COVID-19 pandemic period. CVX data therefore capture ‘normal’ market dynamics as well as distress and recovery periods. The methods yield two cointegrated index series, where the corresponding error correction model can be used as an indicator for market implied tail-risk. Comparing our CVX to existing volatility benchmarks for traditional asset classes, such as VIX (equity) or GVX (gold), confirms that cryptocurrency volatility dynamics are often disconnected from traditional markets, yet, share common shocks.

## Introduction

Since Nakamoto (2008) proposed Bitcoin as a peer-to-peer electronic cash system, this and other cryptocurrencies^1^ have evolved into a new class of financial assets. As of 2021, the crypto domain can be considered one of the most volatile markets available to investors. Volatility, as a measure of the variability of an asset over time, is the most common risk measure in financial theory. We set out to explore volatility and tail-risk in the crypto space by computing benchmarks that are tailored to this young asset class.

Naturally, as cryptocurreny spot markets evolve, markets for derivatives thereon follow. Following the introduction of future contracts, i.e., the mutual *obligation* to exchange some amount of the underlying (e.g., Bitcoin) at a fixed price in the future, investors nowadays have access to option contracts, a type of derivative that gives the buyer the *right* to receive (call option) or deliver (put option) some amount of the underlying for a fixed price (strike) at some future point in time.

Options—like other financial derivatives—are tied to their underlying by an arbitrage relationship, which is based on the replication of the options’s cash-flow. The dynamic nature of an option replication is directly linked to the underlying’s volatility during the lifetime of the contract. Option markets can hence also be seen as markets for volatility. We are interested in extracting volatility information from this unique type of market.

This paper considers two kinds of volatility: first, *historical volatility* that is calculated from previous prices of the underlying; second, *implied volatility* that is inferred from current market prices of options. The latter reflects the market’s expectation of future volatility, in the sense that it is calculated from today’s price for hedging future volatility risk.

A very important benchmark and investment tool are financial indices, which allow investors to obtain information on the current state of the market. Furthermore, indices that are turned into tradable assets and derivatives thereon improve market accessibility. S&P 500 and Euro Stoxx 50, for instance, are two large indices that track North American or European stocks respectively. These price or return indices are complimented by risk benchmarks, most famously CBOE’s Volatility Index (VIX), colloquially dubbed the ‘fear index’, which is designed to capture expected volatility.

In contrast to traditional indices, extracting reliable volatility information from options requires a broad spectrum of high-quality data, which for cryptocurrencies only became available very recently. This is, since cryptocurrency options were introduced in 2016, market liquidity and participation has improved significantly. According to data from Skew^2^, the total number of outstanding contracts (open interest) has more than tripled from its 2019 value, reaching a market size above USD 1 billion for the first time in mid-2020. This surge in size provides a great opportunity to tap a very interesting source of volatility information. Nevertheless, our volatility indexing method addresses remaining liquidity concerns for this young asset class, ultimately allowing us to extract stable cryptocurrency volatility information.

Few attempts have been made so far to implement indices for cryptocurrencies. Trimborn and Härdle (2018) propose a market capitalization weighted crypto-index (CRIX), which as of 2020 tracks 10 cryptocurrencies. With their VCRIX, the authors also propose a volatility benchmark for crypto-assets (Kim et al., 2019). The latter is based on price data and constructed using a heterogeneous autoregressive (HAR) model. As such, the index methodology is different from typical (implied) volatility indices (e.g., VIX) that are based on option prices. The VCRIX is a true index in the sense that it is indexed to a value of 1,000 as of its introduction on 2014-11-28. Most implied volatility indices, in contrast, are not indexed and therefore provide an ad-hoc value for implied volatility.

The research on the volatility of crypto-assets is dominated by questions on portfolio risk, such as the assessment of this new asset class’s potential for portfolio diversification or hedging. By analyzing historical price data, those studies often conclude that cryptocurrencies, despite a considerable speculative component (Fry & Cheah, 2016), bear potential for portfolio diversification and hedging (Akhtaruzzaman et al., 2019; Baur et al., 2015; Bouri et al., 2017a, 2017b; Corbet et al., 2018; Dyhrberg, 2016; Platanakis & Urquhart, 2020). This positive view is challenged, at least in part, by Klein et al. (2018) who claim that Bitcoin is “no safe haven and offers no hedging capabilities for developed markets”. Similarly, Bouri et al. (2018) find spill-over effects between Bitcoin and other assets, “particularly commodities, and therefore, [that] the Bitcoin market is not isolated completely”.

At this point, it is worth mentioning that this paper often uses Bitcoin as a pars pro toto for the entire cryptocurrency market. This has predominantly practical reasons as Bitcoin dominates in liquidity, especially for derivative markets. Our view is backed by the literature that finds strong interdependence within the cryptocurrency market (Ciaian et al., 2018; Corbet et al., 2019). Overall, the literature agrees that, as of now, cryptocurrencies show strong interdependence among each other, however, remain somewhat isolated from the dynamics of traditional markets. A recent study by Giudici and Polinesi (2019) extends this view by stating that “Bitcoin exchange prices are not affected by classic asset prices, but their volatilities are, with a negative and lagged effect”.

In an approach to model return volatility, Katsiampa (2017) explore heteroskedasticity models with regards to Bitcoin price data and find that including both a short-run and a long-run component of the conditional variance (AR-CGARCH) provides the best goodness-of-fit. Similarly, Conrad et al. (2018) use a GARCH-MIDAS model to analyze long- and short-term Bitcoin volatility components and find that S&P 500 realized volatility has a negative and highly significant effect on long-term Bitcoin volatility.

Previous work on cryptocurrency volatility is predominantly concerned with historical volatility, while the literature on implied cryptocurrency volatility is scarce. A major factor in this being that liquid cryptocurrency volatility markets are a very recent development. Some technical articles regarding option pricing models are available (Cretarola & Figà-Talamanca, 2017; Hou et al., 2018), while the improving availability of crypto derivatives data also recently sparks research with a more empirical focus, e.g., Madan et al. (2019) who calibrate advanced option pricing models to Bitcoin options.

Alexander and Imeraj (2020) calculate a Bitcoin volatility index using the CBOE (2019) VIX index methodology. CBOE’s method is the widely recognized market standard for implied volatility indexing and, hence, must be the benchmark and starting point for the development of all volatility indices. Our paper ventures beyond the market standard by acknowledging that the market liquidity of Bitcoin options, even on the most liquid exchanges, is far inferior to the S&P 500 options that are the basis for the original VIX index. We therefore consider alternatives for the volatility extraction as well as index aggregation. The two resulting volatility indices are cointegrated and the corresponding error correction model can be utilized as a metric for market implied tail-risk.

The research on cryptocurrency volatility is scarce and relevant benchmarks that leverage the information available through option markets are hardly accessible to market participants. However, today’s price for hedging future volatility is an important dimension and valuable source of information. In this paper, we aim to contribute to the development of stable, transparent, informative, and replicable benchmarks for future cryptocurrency volatility.^3^

The remainder of the paper is structured as follows: Sect. 2 reviews current markets and conventions for cryptocurrency derivatives, Sect. 3 introduces the index methodology and rules, Sect. 4 presents empirical data for the index, and Sect. 5 concludes.

## Markets and conventions

Before formalizing the index and its rules, this section reviews the underlying market of cryptocurrency derivatives. This is an important exercise as several modelling choices depend on the specifics of the market. This includes, in particular, market liquidity that poses a bigger concern for cryptocurrency derivatives than for most traditional derivative markets.

Market liquidity in our sense is the readiness of participants to exchange the underlying asset and its derivatives. Derivatives are tied to their underlying by an arbitrage relationship. The latter relies on replication, e.g., the replication of an option payout trough dynamic hedging in the underlying. A lack of liquidity leads to unstable and intransparent prices, which in turn limit our ability to assess the fair value of a position (mark-to-market), manage risk, and ultimately trade at a fair price.

**Table 1:** Option contract specifications

	Deribit (BTC)	CME (BTC)	CBOE (SPX)
Underlying	Deribit BTC index	CME BTC (BRR)	S&P 500 index
Settlement	Cash	Cash	Cash
Style	European	European	European
Expiry range	1D–12M	1D–24M	1D–60M
Strike price intervals	250–5000 USD	100–10,000 USD	5 (25) points

Let us consider three relevant measures for liquidity. First, *trading volume*, which measures the instantaneous liquidity based on the number of executed trades in a specific time window (e.g., 24 h); second, *open interest*, which measures the total number of outstanding derivative contracts, regardless of the transaction timestamp; third, *bid-ask spread*, which can be understood as a transaction cost and hence, an entry barrier to the market. According to data from Skew^4^, the majority of cryptocurrency option trading takes place on Deribit^5^, with a market share above $80\%$ (based on average open interest) as of April 2020, followed by LedgerX, OKEx, CME, and Bakkt.

For the purpose of this paper, we follow the liquidity and focus an Deribit and data therefrom. However, market mechanics and contract specifications are similar across all major crypto derivative exchanges. Table 1 provides exemplary option contract specifications from Deribit, CME, and CBOE. Exchange mechanics are also roughly identical; in particular, every exchange that offers derivatives also provides a clearing service that requires an extensive infrastructure on top of the matching engine.

*Clearing* is a mechanism to mitigate counterparty credit risk through a margining system, where a *variation margin* is exchanged to cover mark-to-market changes and an *initial margin* is pledged as a buffer to cover losses from the time where the inability to maintain the variation margin is discovered, to the point where the position is closed. Losses that exceed the available capitalization of a trader have to be covered by an ‘insurance fund’. Such losses from bankrupt traders enter the insurance fund on a regular basis, ranging from a few occurrences per day to a few hundred, e.g., 391 on March 13 2020 during a large spike in Bitcoin volatility.^6^

**Fig. 1:**
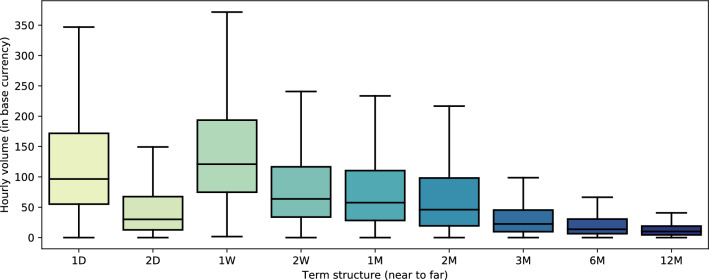
Average hourly option trading volume on Deribit by term structure nodes. Starting from the near term (closest expiry) to the far term. Excluded outliers range up to 1.000 hourly volume in base currency

**Fig. 2:**
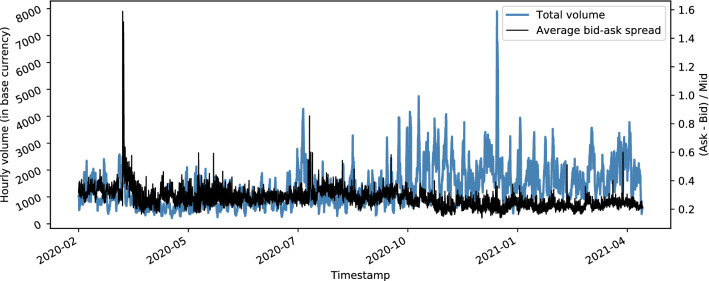
Time-series of total hourly option trading volume and percentage bid-ask spread on Deribit

Options on Deribit are indentified by a symbol that consist of ‘underlying-date-strike-C/P’, e.g., ‘BTC-27MAY20-8750-P’ for a Bitcoin put option with a strike of USD 8750 that expires on 2020-05-27. The options have a multiplier of one, i.e., one option represents the right to buy/sell exactly one BTC at expiry; however, the minimum order size is 0.1 option contracts. Fees on Deribit are currently flat at 0.04% of the underlying or 0.0004 Bitcoin per options contract. On top, the option trader incurs a mean bid-ask spread of 30.2% (8.4% standard deviation) over all strikes and maturities (see Fig. 2). As a reference point, Muravyev and Pearson (2015) observe bid-ask spreads with a mean of 8.6% (4.9% standard deviation) for options on S&P 500 stocks.

To reduce pricing risks and avoid market manipulation, the contractual underlying of cryptocurrency options is often a *spot price index* that averages prices from multiple exchanges. This multi-exchange spot index method addresses the comparably low liquidity on crypto exchanges and is not typically found in option contracts on traditional assets. To reduce settlement risks, a price smoothing procedure is used right before expiry of the option. Such a smoothing mechanism is found in the settlement procedures of many financial derivative. In the example of Deribit, the *exchange delivery settlement price* (EDSP) is calculated using the average of the spot price index over a period of 30 min proceeding expiry. The resulting amount is immediately cash settled in the currency of the underlying.

Figure 1 shows the trading volume for different term structure nodes on Deribit. The term structure is not evenly spaced; the first two nodes are short-term options with 1 and 2 days to maturity. Term 1W and 2W are weeklies, i.e., end of this and next week expiries. The terminal node expires up to 12 month away from spot. Trading volume is not evenly spread over all nodes in the term structure. Prices of far expiry nodes, e.g., 6M and 12M, cannot be expected to be as meaningful and stable as for 1D or 1W. It is worth mentioning that this is a 24/7 option market, with fluctuations in liquidity over time (see Fig. 2).

## Index methodology and rules

The fundamental idea of volatility indices dates back to Brenner and Galai (1989), who envisioned financial instruments for the hedging of volatility changes. To measure such a market price for volatility, we are interested in the implied volatility for an *‘ideal’ at-the-money option with exactly 30 days to maturity*. As such an option is not observable, this section lays out a methodology to extract the ideal option from related option contracts. The method generally applies to all crypto-assets, as long as there exists a liquid option market. Yet, the paper focuses on Bitcoin, due to the currently superior liquidity in Bitcoin options.

The index is designed under three core considerations. First, all meaningful indices in financial markets need to be transparent. Second, if one were to trade the index itself or derivatives thereon, the index must be physically replicable through a portfolio of liquid financial assets. Third, an index must be informative, i.e., represent the desired information about the underlying asset. The latter also implies that the index is comparable to existing volatility indices, such as VIX or other members of the well-established CBOE volatility index family.^7^

Section 3.1 lays out general index rules, such as option selection criteria and the interpolation method. Those index rules are designed to be as similar to existing volatility indices as possible, while accounting for the specifics of cryptocurrency markets. Sections 3.2 and 3.3 introduce two alternative volatility measures that are suitable for the index.

### Selection of option contracts and aggregation

To ensure that only qualified market prices enter the index, all entries with a trading volume of 0 and or without a mid-price are excluded. It is customary that the exchange, through its network of market makers, quotes a ‘mark-price’, even when no trading takes place. Those mark-prices are excluded under the current rules as they do not necessarily reflect actual supply and demand.

In the original CBOE (2019) VIX method, only options with maturity between 23 and 37 days are considered. As options on the S&P 500 trade in biweekly frequency, there are always exactly two expiries considered in the VIX, rendering a simple linear interpolation feasible. Unfortunately, cryptocurrency options do not trade at such granular frequencies. Furthermore, liquidity as measured in terms of trading volume is not spread evenly across expiries (cf. Fig. 1). Choosing exactly two expiries around the target maturity of 30 days might yield unstable results. Therefore, to access additional liquid nodes in the term structure, all expiries between 2 and 60 days from each timestamp are included.

We use *inverse distance weighting* (IDW) for all interpolation tasks, which gives us the flexibility to include additional expiries.^8^ Consider *N* known data points, e.g., expiry–variance pairs, represented as tuples $\{(x_1, u_1), (x_2, u_2), ...,(x_N, u_N) \}$. At an arbitrary point *x*, the interpolated value *u*(*x*) is given by IDW as

1 $$\begin{aligned} u(x) = \sum _{k=1}^N \underbrace{ \frac{ d(x, x_k) ^{-p} }{\sum _{j=1}^N d(x, x_j) ^{-p}} }_{\text {weight}} u_k,\ \ \ \forall \ d(x, x_j) \ne 0, \end{aligned}$$

where the weight is decreasing with an increasing distance $d(x, x_j) = \Vert x-x_j \Vert$. A relevant example for a multi-dimensional interpolation is a volatility surface, which naturally spans over a range of strike prices and maturities. Parameter $p \in {\mathbb {R}}^+$ modifies the weight decay, i.e., a larger *p* leads to a faster weight decay and, hence, to a larger influence of observations that are close to the interpolated point. It is theoretically possible that the target and actual expiry fall on the same date and time and hence have $d(x, x_j) = 0$; in this case, we set $u(x) = u_j$.^9^ Linear interpolation is a special case of IDW with exactly two known data points and $p = 1$. The remainder of the paper uses $p=1$, to remain comparable to existing volatility indices.^10^

The *day count convention* of the index is actual/365, more specifically, as options expire at 08:00 on their expiry date, we calculate option maturity in minutes to expiry for each observation timestamp and divide by 525,600 = 365 × 1440, the total number of minutes in a 365-day year. The resulting act/365 time-to-maturity is henceforward referred to as $\tau$.

Brenner and Galai (1989) envisioned that market participants would be able to hedge their exposure to changes in volatility by trading volatility itself. Our index lends itself to be used as such a tradable volatility asset and hence, also as an underlying for volatility futures and options. However, the currently relatively large and varying number of strikes and expiries entering the index increases complexity and, thus, makes it harder to physically replicate the index. That being said, decreasing the number of assets by focusing on fewer nodes would increase concentration risk and make the index more susceptible to manipulation. Considering that the young cryptocurrencies derivative market cannot provide the liquidity that is available on mature markets, we decided in favour of diversification over a larger number of strikes and expiries. This assessment might be updated once the liquidity situation for cryptocurrency derivatives improves.

### Model free volatility (CVX)

Britten-Jones and Neuberger (2000) pioneered a risk-neutral variance forecast that does not rely on a specific model, but only on market prices for option contracts. Their method is applied to most modern volatility assets, such as variance swaps or the VIX volatility index family.^11^

A variance swap is an over-the-counter derivative for the forward exchange of squared realized volatility $\sigma _\mathrm {real}^2$ of some underlying (e.g., S&P 500) at an ex-ante defined variance swap level (strike) *v*. At expiry, the difference $(\sigma _\mathrm {real}^2 - v) \times A$ is settled in cash, where *A* is the notional amount of the swap per annualized volatility point. The realized volatility is conventionally calculated on closing price log-returns.

The theory developed in Britten-Jones and Neuberger (2000), Carr and Madan (2001) and Demeterfi et al. (1999a), who assume essentially only a positive and continuous price path for the underlying asset yields a self-financing strategy that replicates the continuously observed $\sigma _\mathrm {real}^2$ for a non-dividend-paying asset. Carr and Lee (2007) call this replicating portfolio, which only requires a static position in options^12^ and dynamic position in the underlying asset, a synthetic variance swap.

The replication of variance swaps enables us to determine the ex-ante fair swap level $v^*$. The swap is fair if at inception $E[\sigma _\mathrm {real}^2 - v^*] = 0$, hence, today’s $v^*$ is the market implied future variance, i.e., the quantity that we want to track with our CVX index. As laid out in detail by Demeterfi et al. (1999b), the annualized variance swap level is

2 $$\begin{aligned} v^* = \frac{2e^{r \tau }}{\tau } \left( \int _0^F \frac{1}{K^2} P(K) dK + \int _F^\infty \frac{1}{K^2} C(K) dK \right) , \end{aligned}$$

with prices for put *P*(.) and call *C*(.) options, strike *K*, current forward price of the underlying *F*, and risk-free interest rate *r* for maturity $\tau$. Note that Eq. (2) is a special case of the variance swap equation^13^, where *F* is both the forward level as well as the cut-off between put and call prices.

The fair variance swap level *v* is based on a continuum of strikes *K*; in practice, there exists a finite set of ordered strikes $K_i \in {\mathbb {R}}^+$. To ease notation, strikes below the forward level *F* are indexed with $i \in \{1, 2, ..., n\}$ and associated with mid-prices for put options $P(K_i)$. Conversely, call options where $K_i > F$ are index with $i \in \{n+1, ..., N\}$. The only exception to this out-of-the-money principle is the case where $K_i = K_n$, i.e., the first strike below the forward level, where $P(K_i)$ is the average mid-price of both put and call.

The following Riemann sum approximates Eq. (2) and is identical to the method underlying the CBOE (2019) VIX index family. The quantity of interest that enters the volatility index is the market implied variance, which is calculated from options with maturity $\tau$ as

3 $$\begin{aligned} \sigma ^2_\tau&= \frac{2 e^{r \tau }}{\tau } \left( \sum _{i=1}^n \frac{\varDelta K_i}{K_i^2} P(K_i) + \sum _{i = n+1}^N \frac{\varDelta K_i}{K_i^2} C(K_i) \right) \underbrace{- \frac{1}{\tau } \left[ \frac{F}{K_n} - 1 \right] ^2}_{\text {ATM adjustment}}, \end{aligned}$$

with strike interval

$$\begin{aligned} \varDelta K_i&= \frac{K_{i+1} - K_{i-1}}{2}, \quad \text {for } 1< i < N. \end{aligned}$$

The intervals on both ends of the strike range are $\varDelta K_1 = K_2 - K_1$ and $\varDelta K_N = K_N - K_{N-1}$, respectively.

It is unlikely that any discrete $K_i$ coincides with the continuous *F*; however, the swap pricing formulae requires that $K_n = F$. The ATM adjustment therefore accounts for the distance between the forward level *F* and the first strike below the forward level $K_n$. This is, the forward adjustment shifts the strikes to their required at-the-money levels.

Equation (3) yields the implied at-the-money variance $\sigma _{\tau }^2$ for maturity $\tau$. In a final step, these variances have to be interpolated over their respective maturities, because the CVX is designed to reflect the at-the-money volatility for exactly 30 days. For each $\tau$, the maturity distance to the ideal option can be written as

4 $$\begin{aligned} d(\tau ) = \sqrt{(30/365 - \tau )^2}. \end{aligned}$$

Using the interpolation from Eq. (1), where $u_k = \tau \sigma _{t,\tau }^2$ and $d(x, x_j) = d(\tau )$, the interpolated annualized volatility is the index level at time *t*

5 $$\begin{aligned} \mathrm {CVX} = \sqrt{\sigma ^2 \times 365/30} \times 100. \end{aligned}$$

Despite its popularity, the literature discusses shortcomings of the method, especially in lieu of heavy-tailed markets. Choi and Yang (2019) show empirically that the approximation error under a jump diffusion process can be as much as 5%; however, the authors also find that the error for the majority of their data is below $1\%$. Similarly, Chow et al. (2018) claim that VIX undervalues volatility when returns are expected to be negatively skewed and vice versa. Said authors propose an alternative method (‘GVIX’) that aims at resolving these shortcomings. However, for the purpose of this paper, comparability to existing benchmarks outweighs technical improvements.

### Model implied volatility (CVX76)

In the simplest of option pricing models, volatility is the only free parameter that is not observable on the market. Once market prices for options become available, one can use said models to extract implied information by solving for volatility. There exists a plethora of more advanced option pricing models, with an interesting exercise in financial engineering research being to ‘horse-race’ models to find the best fit for a given underlying. Madan et al. (2019) provide such a study for cryptocurrency options. For the purpose of a volatility index, however, it is paramount that the only information extracted, i.e., the only free parameter in the model, is implied volatility. This excludes many advanced option pricing formulae and brings us back to plain vanilla Black–Scholes type models. More specifically, this paper uses the Black (1976) model to compute the implied volatility from a market of cryptocurrency options and futures. This is similar to the implied volatility—sometimes called ‘Black’ volatility—that is available, e.g., through Bloomberg’s volatility functions.

With forward price of the underlying *F*, for maturity $\tau$, risk-free interest rate^14^*r*, strike *K*, and (implied) volatility $\sigma$, the price of a European call option is

6 $$\begin{aligned} C&= \left[ F \varPhi (d_+) - K \varPhi (d_-) \right] e^{-r \tau }, \end{aligned}$$

and for the put option

7 $$\begin{aligned} P&= \left[ K \varPhi (-d_-) - F \varPhi (-d_+) \right] e^{-r \tau }, \end{aligned}$$

where

$$\begin{aligned} d_\pm = \frac{\ln (F/K) \pm \left( \sigma ^2 / 2 \right) \tau }{\sigma \sqrt{\tau }}, \end{aligned}$$

and $\varPhi (.)$ is the cumulative normal distribution function.

Extracting implied volatility from a market of option prices is not possible in closed form, hence, a Newton–Raphson (NR) algorithm is used for the job.^15^ Besides the pricing model in Eqs. (6) and (7), the algorithm requires the first-order sensitivity with respect to implied volatility, a quantity that is know to option traders as ‘Vega’

$$\begin{aligned} \mathrm {Vega} = \frac{\partial c}{\partial \sigma } = F \sqrt{T} \varPhi (d_+) e^{-r \tau }. \end{aligned}$$

The NR algorithm is used to compute the volatility surface for each timestamp in the sample. This leaves us, for every point in time *t*, with a surface of implied volatilities $\sigma (\tau , K)$ that spans over all strikes *K* and maturities $\tau$ of the available options.

The index is designed to represent the implied volatility of a 30 days to maturity at-the-money option. The surface is interpolated accordingly by inverse distance weighting as introduced in Eq. (1), where the distance to the target option at time *t* is

$$\begin{aligned} d(\tau , K) = \sqrt{(30/365 - \tau )^2 + (F - K)^2}. \end{aligned}$$

The interpolated volatility is the index level at time *t*

8 $$\begin{aligned} \mathrm {CVX76} = \sqrt{\sigma ^2} \times 100. \end{aligned}$$

The first and foremost shortcoming of this method is the model itself, which requires a number of limiting assumptions. Most importantly, the Black 76 model assumes normally distributed log-returns, an assumption that is not warranted for financial assets in general and cryptocurrencies in particular. In a normally distributed world, both CVX and CVX76 should produce very similar results. However, it is well established that (cryptocurrency) returns are heteroskedastic and heavy-tailed (Osterrieder & Lorenz, 2017), and thus, experience returns that are larger and in higher frequency than expected under a normal distribution. We will revisit the differences between CVX and CVX76 when analyzing the empirical data in the following section.

## The cryptocurrency volatility index (CVX)

Data are available and kept up-to-date at www.thecvx.com.

### Data

We collect data snapshots in 5 min intervals for all option and future contracts that are traded on Deribit. The data are available from February 6, 2020 until July 6, 2021. In addition, we collect intra-day data for CBOE’s volatility indices^16^ from Reuters Eikon, namely:

- VIX: is designed to measure 30-day expected equity volatility. The index is derived from mid-quote prices of S&P 500 index options, as such it is closely related to the CVX that was introduced in Sect. 3.2.
- RVX: is a measure of 30-day expected equity volatility similar to VIX, however, with Russell 2000 index options as underlying.
- VVIX: measures expected volatility of the 30-day forward price of the VIX, and hence, the index represents the volatility of volatility. VVIX is calculated from mid-quote prices of VIX index options using the same method used to calculate the VIX index itself.
- GVX: is designed to measure 30-day expected volatility of gold prices. The index is calculated by applying the VIX methodology to options on SPDR Gold Shares, an ETF representing gold bullion held by the SPDR Gold Trust.
- EUVIX: 30-day expected EUR/USD exchange rate volatility from FX options.
- TYVIX: 30-day expected volatility of U.S. Treasuries. The index is calculated from futures options on 10-year Treasury Notes (ticker TY), with a methodology similar to VIX.
- SRVIX: 1-year implied volatility of the 10-year swap rate. The index is calculated from 1-year swaptions on 10-year USD interest rate swaps.

A concise overview is provided in Table 2.

**Table 2:** Volatility indices used in this paper

Symbol	Underlying	Time-range	Methodology	Data since
CVX	Bitcoin	30-day	Model-free	2020
CVX76	Bitcoin	30-day	Black 76	2020
VCRIX	CRIX	30-day	HAR model	2014
VIX	S&P 500	30-day	Model-free	1990
RVX	Russell 2000	30-day	Model-free	2004
VVIX	VIX	30-day	Model-free	2006
GVX	COMEX Gold futures	30-day	Model-free	2011
EUVIX	EUR/USD FX	30-day	Model-free	2007
TYVIX	10-year Treasury Notes futures	30-day	Model-free	2007
SRVIX	10-year swap rate	1-year	Model-free	2012

VCRIX is calculated from return data, whereas all other indices are based on option price data

Siriopoulos and Fassas (2019) provide a more complete overview of volatility indices for traditional financial assets

### Results

**Fig. 3:**
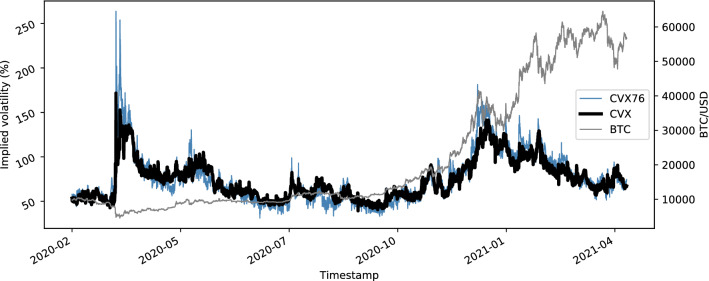
Annualized 30-day expected volatility for Bitcoin in hourly frequency. Note the volatility spike on Friday the 13 of March 2020, the day when New York City started to lock down due to the COVID-19 outbreak

Figure 3 shows the expected Bitcoin volatility in hourly frequency as captured by CVX and CVX76. CVX is the model-free annualized expected volatility over the next 30 days, which is based on mid-prices for Bitcoin options (see Sect. 3.2). CVX76 is based on the Black 76 model implied volatility and interpolated from a volatility surface for each timestamp in the data (see Sect. 3.3). Both indices are based on the same option data.

*The volatility indices CVX and CVX76* often appear inversely related to their underlying. This is a typical observation for volatility indices. On one hand, market corrections are typically more rapid than upward moves and cause spikes in volatility. On the other hand, with a net long position over all investors in the underlying, the demand for downside protection drives volatility prices especially when markets are falling.

**Fig. 4:**
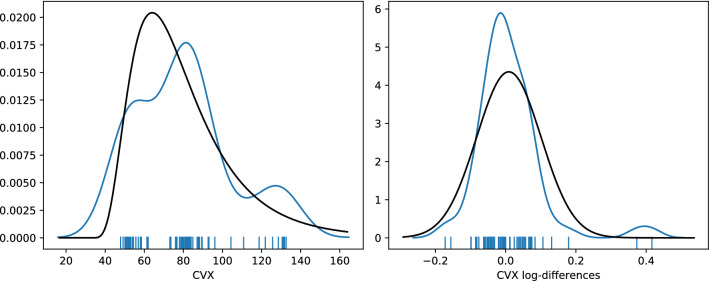
Gaussian kernel density estimate and rug plot (blue). Left: CVX with fitted log-normal distribution. Right: CVX log-differences with fitted normal distribution (color figure online)

Figure 4 shows the Gaussian kernel density estimate for CVX and CVX log-differences as a blue line. The black line indicates a log-normal (left) and normal (right) distribution fit. A rug plot just above the x-axis indicates the frequency and clustering of observations. One can clearly see the two main clusters of observations for CVX, i.e., a pre-COVID-19 regime around 50–60 and post-regime around 75–85.

It is well established that asset returns, especially crypto-assets (Osterrieder & Lorenz, 2017), are heteroskedastic, heavy-tailed, and susceptible to jumps. Similar dynamics can be observed for our cryptocurrency volatility indices. This has interesting implications, especially for the CVX76, as the index methodology relies on the assumption of normally distributed log-returns in the underlying, which is frequently challenged by strong market movements. More specifically, when comparing the index data of CVX and CVX76, one can see that the indices are more similar during less volatile times and vice versa. We want to further investigate these joint dynamics before returning to the analysis of cryptocurrency volatility.

**Fig. 5:**
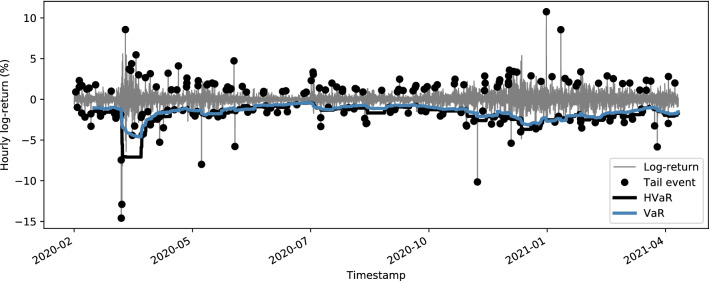
Bitcoin returns and value at Risk. Black dots indicate tail events that are defined as returns where the standardized residual of a GARCH(1,1) process is above (below) the 99% (1%) quantile

*Differences between CVX and CVX76* are obviously owed to the fundamentally different calculation methods; however, both methods are in principle designed to quantify the same information, i.e., 30-day market implied volatility. It appears that CVX76 is similar to CVX in ‘normal’ markets, but produces much more extreme results during times of strong market movements. We present evidence that both indices are cointegrated and that CVX76 tends to return to the level of CVX—not vice versa—which supports the argument that

1. CVX is a more accurate representation of market expected volatility,
2. the deviation of the indices is caused by the CVX76 normality assumption, and
3. the $(\mathrm {CVX} - \mathrm {CVX76})$ holds information on market implied tail-risk.

In a nutshell, out-of-the-money option prices, especially during strong market moves, are higher than suggested by a light-tailed normal distribution. This effect is compensated by a particularly high Black-76 implied volatility. Recall that the CVX76 is constructed as a measure for at-the-money volatility, by interpolation over a range of strikes, including out-of-the-money options. We postulate that both indices share a strong relationship that is sometimes distorted, especially during large movements in the underlying, but subsequently corrected. This claim is corroborated by the following cointegration analysis.

We follow the Engle and Granger (1987) two-step method to analyze the joint behaviour of CVX and CVX76. First, we use the Augmented Dickey–Fuller (ADF) test^17^ to confirm that the differences as well as the log-differences of CVX and CVX76 are stationary, and hence, the two time-series are integrated of order 1, in the sense of cointegration. Second, we estimate the cointegrating regression

$$\begin{aligned} \mathrm {CVX76}_t = \beta \ \mathrm {CVX}_t + \epsilon _t, \end{aligned}$$

and test $\epsilon _t$ for stationarity using the ADF test. A t-statistic of 5.856 confirms that CVX and CVX76 are indeed cointegrated. The strong relationship implies that distortions to the long-term equilibrium of both indices are temporary and correcting over time.

The adjustment behaviour can be analyzed by estimating the underlying error correction model. This is, given a distortion of the equilibrium level, a change in the index $\varDelta \mathrm {CVX76}_t$ should include an adjustment component towards the equilibrium level. The error correction model is specified as

9 $$\begin{aligned} \varDelta \mathrm {CVX76}_t = \alpha \ \epsilon _{t-1} + \gamma \ \varDelta \mathrm {CVX}_t + u_t, \end{aligned}$$

where $\varDelta$ is the first difference operator, $u_t$ is i.i.d., and $\epsilon _{t-1}$ can be interpreted as the equilibrium error in the previous period. If the error is 0, the model is in equilibrium and vice versa. Both $\alpha$ and $\gamma$ capture short-term dynamics; however, the actual equilibrium error adjustment is captured by parameter $\alpha$. In this model specification, a negative $\alpha$ implies that the $\mathrm {CVX76}$ moves back towards $\mathrm {CVX}$, until the equilibrium relationship is re-established.

We find the adjustment parameter (*t*-statistic) to be statistically significant at − 0.0475 (− 5.285). Finally, we want to estimate how long it takes for an existing error to be reduced by half, i.e., the half-life of the disequilibrium. Solving for the number of periods *n* in $(1 + \alpha )^n = 50\%$ yields a half-life of $n = 14.25$. Recalling that the indices are calculated in hourly frequency leaves us with a half-life of roughly 14 h. Swapping the indices in Eq. (9) does not yield a negative adjustment coefficient, and hence, $\mathrm {CVX76}$ adjusts towards $\mathrm {CVX}$ but not vice versa.

Changes in the spread between both indices, i.e., deviations from the equilibrium, provide information as an indicator of *market implied tail-risk*. That is, the indices diverge in markets where a normal distribution is not able to reflect the actual price movements, i.e., a heavy-tailed market environment. A similar tail-risk metric, which is based on GARCH models with normal and heavy-tailed innovations, has previously been applied to construct tail-risk protection strategies (Packham et al., 2017).

We make the following observations with respect to the spread between both indices that we define as $\mathrm {76spread}_t = (\mathrm {CVX76}_t - \mathrm {CVX}_t) / \mathrm {CVX}_t$. First, there exists a strong correlation between the $\mathrm {76spread}$ and negative tail returns^18^ (see Fig. 5), with a correlation coefficient of − 0.68. In comparison, the correlation with all returns is merely − 0.05. Second, there exists a positive correlation, yet less strong (0.14), between $\mathrm {76spread}$ and changes in the Value at Risk (VaR)^19^, which also persists when lagging the $\mathrm {76spread}$. These correlations could hint at the premium that investors are willing to pay for future tail-risk, an argument that has already been made for Equity markets (Bollerslev & Todorov, 2011). However, additional tests would be necessary to further substantiate this claim in the context of Cryptocurrencies.

**Fig. 6:**
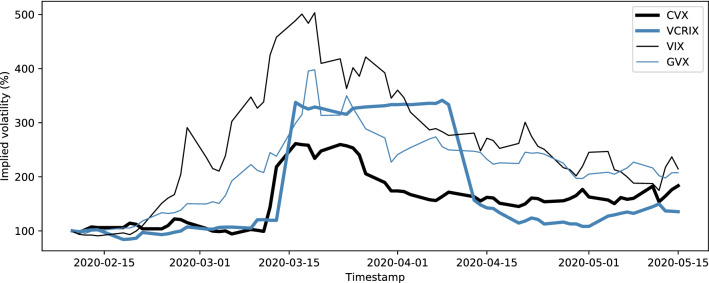
Implied volatility for Bitcoin, S&P 500 and gold, indexed to 100 on 2020-02-10. It appears that Bitcoin volatility starts reacting to the COVID-19 shock about 1 month later than equity and gold

*Compared to classical asset volatilities*, cryptocurrency volatility dynamics are often disconnected, yet, share common shocks. Figure 6 shows that Bitcoin was slow to react to the overall market distress that was caused by the COVID-19 crisis, which transmitted to cryptocurrencies roughly 30 days after traditional assets already experienced a sharp increase in expected volatility. This supports the results in Alexander and Imeraj (2020), who examine the co-movement of Bitcoin variance and its risk premium with traditional assets, thereby showing that, after the outbreak, “Bitcoin itself and also its variance has been behaving very similarly to traditional assets”. The results are also similar to the paper on *realized volatility* from Conrad et al. (2018), where the authors find that equity volatility has a delayed spill-over effect on cryptocurrency volatility.

**Fig. 7:**
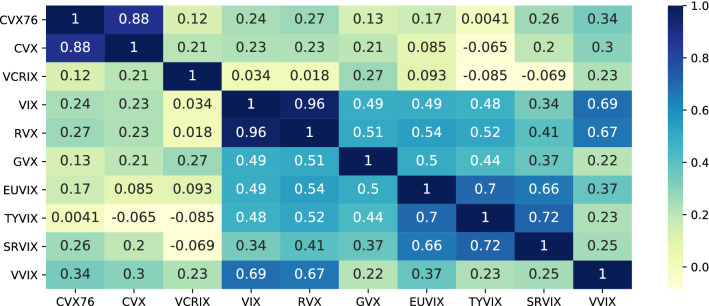
Heat-map of Pearson correlations between daily log-differences of implied volatility indices. Cryptocurrency volatility appears disconnected from the cluster of traditional asset correlations. Both CVX and VCRIX measure cryptocurrency volatility, but use fundamentally different index methodologies, hence, the very low correlations

The VCRIX in Fig. 6 is an econometric cryptocurrency volatility benchmark, proposed by Trimborn and Härdle (2018). It is based on price data and constructed using a heterogeneous autoregressive (HAR) model. As such, the index methodology is fundamentally different from our implied volatility indices, which are based on option prices. The VCRIX is a true index in the sense that it is indexed to a value of 1, 000 as of its introduction on 2014-11-28. In contrast, all other implied volatility indices considered in this paper are not indexed but rather provide an ad-hoc value for implied volatility. The indices in Fig. 6 have been re-index to a basis of 100 as of 2020-02-10, to make them comparable.

The heat-map in Fig. 7 shows Pearson correlations between log-differences of major volatility indices. The correlation between Bitcoin and other volatility ranges roughly between 0.1 and 0.3, whereas classical assets show higher correlations. The disconnection from the dynamics of traditional markets supports claims on the potential for portfolio diversification made by, e.g., Baur et al. (2015), Bouri et al. (2017a, 2017b) and Dyhrberg (2016). Both CVX and VCRIX measure cryptocurrency volatility, but use fundamentally different index methodologies, hence, the low correlations.

**Fig. 8:**
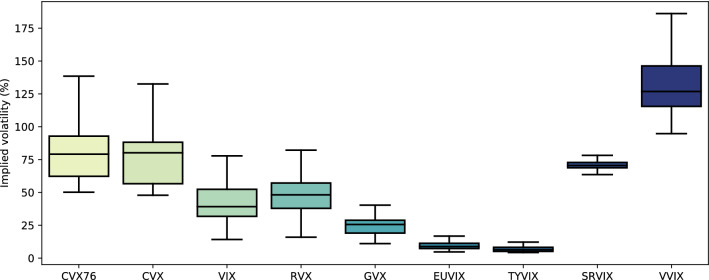
Box-plots of annualized implied volatility for Bitcoin, S&P 500, Russel 2000, VIX, gold, crude oil, EURUSD FX, US treasuries, and interest rate swaptions

It is well established that asset return correlations are not constant over time and may be strongly affected by specific events, hence, present an important risk factor.^20^ Engle and Figlewski (2015) provide similar evidence on the dependence among implied volatilities. This has important implications for hedging. In a nutshell, the prices for hedging increase when protection is needed most. This makes the somewhat disconnected dynamics of cryptocurrencies particularly interesting.

The current research that attributes great diversification potential to crypto-assets is based on the presumption that these assets remain an exotic asset class with dynamics that are separated from traditional markets. While this claim is currently supported by the data, increasing acceptance of this market might drive overall market integration in the future and in turn bring crypto market dynamics closer to traditional assets. The COVID-19 crisis already showed that cryptocurrencies, despite their delayed response, are subject to systemic volatility shocks.

Box-plots in Fig. 8 show that—unsurprisingly—expected volatility is usually higher for cryptocurrencies than traditional assets. The two exceptions are volatility of volatility (VVIX) and crude oil volatility (OVX, not-shown). The first is naturally an extremely volatile asset class. The latter recently saw its highest levels since inception in 2008, which was primarily driven by the 2020 oil price war between Russia and Saudia Arabia and is therefore of no further interest to this study.

## Conclusion

Since the invention of Bitcoin, cryptocurrencies have evolved into a new class of financial assets. Naturally, as cryptocurrency spot markets evolve, markets for derivatives thereon follow. Of those, option markets offer the unique potential to extract volatility information that would otherwise be unobservable. We extract said information through a cryptocurrency volatility index (CVX) that captures the market’s expectation of future volatility.

Volatility is an important metric and the most common risk measure in finance. Accessing stable and reliable volatility information is of fundamental interest to investors and risk managers alike. However, implied volatility must be based on a broad spectrum of liquid and reliable option prices, and hence, requires a much larger data foundation than realized volatility. Our method addresses liquidity concerns for this young asset class by broadening the base of relevant options, when compared to volatility benchmarks for traditional assets (e.g., VIX). Given this method, we find that the liquidity on cryptocurrency option exchanges is sufficiently developed to produce stable results. This also means that, despite being calculated 24/7 and only with the information and liquidity available at any given point in time, the CVX appears smooth and reflective of the underlying.

Comparing the volatility dynamics captured by CVX to traditional volatility benchmarks, we observe that cryptocurrencies live a somewhat secluded life and therefore bear diversification potential, a finding that is in line with the literature. However, despite a lag, the COVID-19 crisis is a good example for a global shock that affects cryptocurrencies and traditional assets alike. This is additional evidence on the limits of diversification during times where it is needed most.

The model-free CVX index should yield a better estimate for markets’ expected volatility than the CVX76. However, due to the assumption of normally distributed log-returns in the Black-76 method, the $(\mathrm {CVX} - \mathrm {CVX76})$ spread is an interesting indicator of market implied tail-risk. More specifically, the two indices share the strong statistical bound of cointegration, which is temporarily distorted during heavy-tailed markets. An error correction model shows that said distortions have an average half-life of roughly 17 h. This gives an indication of the time it takes for this market to ‘normalize’.

Cryptocurrency option liquidity is centred on Bitcoin, which is currently a limit to the accessibility of cryptocurrency volatility. Until liquidity spreads out to other assets, Bitcoin has to be used as a surrogate for the entire asset class. Preferably, a liquid option market on an index such as the CRIX could be used in future to significantly improve the scope of the CVX, without the risk of fragmented liquidity in the underlyings. This would ultimately provide two very interesting perspectives on volatility, namely, the econometric VCRIX and the market implied CVX.

## Footnotes

1 For an introduction to Cryptocurrencies, i.e., digital assets that use a distributed ledger or blockchain technology, we refer to Härdle et al. (2019).

2 Skew is a London-based cryptocurrency data provider; see: www.skew.com/dashboard/bitcoin-options.

3 Our indices are available and kept up-to-date at www.thecvx.com.

4 Skew: www.skew.com/dashboard/bitcoin-options.

5 Deribit is a cryptocurrency derivatives exchange that operates since 2016, see: www.deribit.com.

6 Deribit states that *“the 12–13 March 2020 extreme market volatility had a big impact on the size of the Deribit BTC insurance fund [...].”*, prompting several injections to the fund. See www.deribit.com/main#/insurance (Accessed 18 June 2020).

7 For an overview, see: www.cboe.com/products/vix-index-volatility/volatility-indexes.

8 Furthermore, this multivariate interpolation method allows us to interpolate on the volatility surface, i.e., to interpolate between different strikes and expiries.

9 This case is very unlikely as our intra-day data are collected with a timestamp in millisecond precision. For a multi-dimensional interpolation (volatility surface), this case becomes even more unlikely.

10 CBOE’s aforementioned VIX method uses linear interpolation between a near- and far-term option.

11 See Carr and Lee (2009) for an overview of volatility derivatives and Demeterfi et al. (1999b) for an in depth review of variance swaps.

12 More precisely, a static holding of put options with strikes below an arbitrary cut-off level and call options with strikes above.

13 Equation (26) in Demeterfi et al. (1999b) uses an arbitrary cut-off level $S^*$. This paper uses an at-the-money forward ﻿cut-off level, and hence, $S^* = F = S_0 e^{rT}$, where $S_0$ is today’s price of the underlying.

14 We assume a 30-day risk-free interest rate of 0.

15 Using the Brenner and Subrahmanyan (1988) approximation of implied volatility as initial guess for the search algorithm

$$\begin{aligned} \sigma _0 = \frac{C_\mathrm {atm} \sqrt{2 \pi }}{F \sqrt{\tau }}, \end{aligned}$$

did not provide satisfactory results as the method systematically underestimates implied volatility for our cryptocurrency options, and hence, often fails to converge to the target tolerance within the desired number of iterations. Increasing the initial guess to a constant $\sigma _0 = 1.5$ reduces the number of failed attempts to $0.45\%$.

16 An overview of all CBOE volatility indices is available at www.cboe.com/products/vix-index-volatility/volatility-indexes.

17 We always run the ADF test in 3 settings: with constant, without constant and with trend. Only the least significant test statistic out of all settings is reported in the paper.

18 We account for heteroscedasticity and define negative (positive) tail-events as returns where the standardized residual of a GARCH(1,1) process is below (above) the 1% (99%) quantile.

19 We compute the Value at Risk as a quantile of the BTC return distribution. Figure 5 shows a historical simulation (HVaR) and delta-normal (VaR); both methods are standard in the literature and we refer to Jorion (2009) for technical details.

20 See Longin and Solnik (2001), Packham and Woebbeking (2019), Pu and Zhao (2012) and Wied et al. (2012).
